# Head Growth and Neurodevelopment of Preterm Infants with Surgical Necrotizing Enterocolitis and Spontaneous Intestinal Perforation

**DOI:** 10.3390/children8100833

**Published:** 2021-09-23

**Authors:** Seung-Han Shin, Ee-Kyung Kim, Seh-Hyun Kim, Hyun-Young Kim, Han-Suk Kim

**Affiliations:** 1 Department of Pediatrics, College of Medicine, Seoul National University, Seoul 03080, Korea; revival421@snu.ac.kr (S.-H.S.); drcorkim@gmail.com (S.-H.K.); kimhans@snu.ac.kr (H.-S.K.); 2 Department of Pediatric Surgery, Seoul National University College of Medicine, Seoul 03080, Korea; spkhy02@snu.ac.kr

**Keywords:** necrotizing enterocolitis, neurodevelopment, preterm infants, spontaneous intestinal perforation

## Abstract

Spontaneous intestinal perforation (SIP) and surgical necrotizing enterocolitis (NEC) are intestinal conditions requiring surgical intervention in preterm infants. We aimed to compare the head growth and neurodevelopment of preterm infants with SIP and surgical NEC. A retrospective single-center study was performed in preterm infants born at less than 32 weeks of gestation and who had undergone surgery for NEC or SIP. Data from the Bayley Scales of Infant and Toddler Development 3rd Edition (Bayley-III) at 24 months of corrected age (CA) and the Korean Ages and Stages Questionnaire (K-ASQ) or Korean Developmental Screening Test (K-DST) at 36 months were collected. Among 82 eligible infants, 60 infants had surgical NEC, and 22 infants were diagnosed with SIP. Head growth was faster until CA 4 months in preterm infants with SIP than in those with surgical NEC. At 36 months, abnormal findings in the K-ASQ or K-DST were more prevalent in the NEC group than in the SIP group in the gross motor (48.2% vs. 0%, *p* = 0.015), fine motor (40.7% vs. 0%, *p* = 0.037), cognitive (55.6% vs. 12.5%, *p* = 0.047), and social domains (44.4% vs. 0%, *p* = 0.032). More studies evaluating the neurodevelopmental outcomes of preterm infants with surgical NEC and SIP are required.

## 1. Introduction

Morbidities in preterm infants can disturb the growth and development of the baby, and at times, can cause mortality during or after the neonatal period. Necrotizing enterocolitis (NEC) is one of the most common intestinal morbidities in preterm infants, occurring in 5–10% of very low birth weight infants or very preterm infants [1,2]. A quarter of infants with NEC requires surgical intervention, with an overall case-fatality rate of 15% [3]. Preterm infants with surgical NEC have demonstrated a higher incidence of growth retardation, and a more advanced stage of NEC has been associated with severe growth retardation [4,5]. Moreover, NEC has been associated with neurodevelopmental impairments (NDIs) in preterm infants, especially when surgical intervention is required [5,6,7]. Profound inflammatory response and nutritional deprivation can contribute to poor developmental outcomes in infants with surgical NEC [8].

Spontaneous intestinal perforation (SIP) is another important intestinal disease in preterm infants requiring surgical intervention and is distinct from NEC in its pathophysiology and clinical manifestations [9]. Smaller babies at birth, lower Apgar scores, and earlier disease onset have been associated with preterm infants with SIP than those with surgical NEC. More infants with surgical NEC died than infants with SIP [10,11]. However, there have been controversies regarding the neurodevelopmental outcomes of preterm infants with SIP as compared with those with surgical NEC. Two retrospective studies from the Neonatal Research Network showed that among very low birth weight infants or extremely low birth weight infants, neurodevelopment was comparable between infants with surgical NEC and SIP before 24 months of age [11,12]. However, a retrospective observational study showed that infants with surgical NEC displayed worse developmental outcomes at 1 year of age, accompanied by subnormal head growth [13]. As white matter injury was more evident in preterm infants with surgical NEC, head growth may be different in this population, which could subsequently result in suboptimal neurodevelopment [14,15].

In the present study, we aimed to investigate the growth pattern of preterm infants with surgical NEC and SIP during and after a neonatal intensive care unit (NICU) stay and to compare the neurodevelopmental outcomes at CA 24 and 36 months of age between the two groups.

## 2. Materials and Methods

### 2.1. Study Design and Participants

This retrospective study was approved by the Institutional Review Board of Seoul National University Hospital (2107-057-1233), and the requirement for informed consent was waived. The study protocol was in accordance with the Declaration of Helsinki, and all methods were performed in accordance with the guidelines of the Human Research Protection Program. The study included preterm infants of gestational age (GA) less than 32 weeks who were admitted to the NICU of Seoul National University Hospital and had underwent surgical intervention for NEC or SIP between October 2006 and October 2018. Infants with major congenital anomalies were excluded from the study. NEC was diagnosed based on radiologic findings using modified Bell’s staging criteria and supplemented by operative and pathologic findings [16,17]. SIP was diagnosed when intestinal perforation occurred without any evidence of NEC or intestinal anomalies. The study population was categorized into surgical NEC and SIP groups.

Perinatal characteristics including GA, birth weight, Apgar scores, sex, mode of delivery, use of antenatal steroids, and small for gestational age (SGA) status, were collected from electronic medical records, in addition to clinical findings such as respiratory distress syndrome (RDS), bronchopulmonary dysplasia (BPD), sepsis, surgically treated patent ductus arteriosus (PDA), retinopathy of prematurity (ROP) requiring laser photocoagulation, intraventricular hemorrhage (IVH), and periventricular leukomalacia (PVL). SGA was defined as a birthweight less than the 10th percentile for gestational age and sex according to the Fenton growth charts [18]. Diagnosis and severity of BPD were determined based on the 2001 National Institute of Child Health and Human Development criteria [19]. The grade of IVH was classified based on Papile’s criteria [20]. Perioperative characteristics, such as medications before surgery, weight, and postnatal day at operation, were reviewed. Infants with conditions associated with congenital hearing loss such as congenital cytomegalovirus or congenital anomalies were not included in this study.

### 2.2. Measures and Outcomes

Growth parameters including weight and head circumference at birth, at discharge, at 4 and 24 months of corrected age (CA), and at 36 months of age were collected. Z-scores for sex and age were calculated based on the Fenton growth charts or WHO growth charts [18]. Weekly head circumferences of the study population were reviewed from the postmenstrual age (PMA) of 28 to 34 weeks, and z-scores for sex and PMA were calculated to determine the timing of nadir of head circumference growth. Following this, the z-score change of each growth parameter across time was calculated and compared between the two groups.

Data on developmental outcomes were collected at 24 and 36 months of age. At 24 months of age, the Bayley Scales of Infant and Toddler Development 3rd Edition (Bayley-III) results were reviewed, and scores < 85 (<−1 SD) in both the cognitive and language domains or a motor score < 85 were defined as delays in development [21]. Hearing impairment was defined as the need for unilateral or bilateral hearing aids. Infants with any of the following criteria at a CA of 24 months were defined as having combined NDI: any delay in Bayley-III, cerebral palsy (CP), hearing impairment, or blindness [22]. Additionally, data from the Korean Ages and Stages Questionnaire (K-ASQ) or the Korean Developmental Screening Test (K-DST) at 36 months of age were collected. The K-ASQ was developed based on the ASQ-II and revised for use in Korea [23]. It is composed of five domains (communication, gross motor, fine motor, problem-solving, and personal-social) and contains 30 questions to be answered by the caregivers. The K-DST was developed to replace the K-ASQ in Korea and to evaluate six areas of development (gross motor, fine motor, cognition, language, sociality, and self-care) [24]. As infants were tested using the K-ASQ or K-DST during the study period, abnormal findings in relevant domains from the two screening tools were summed up according to the following items: language, gross motor, fine motor, cognitive or problem-solving, and social domains.

### 2.3. Statistical Analysis

Data analysis was performed using STATA 12.0 for Windows (Stata Corp, College Station, TX, USA). The Wilcoxon rank-sum test was used to compare continuous variables, and Fisher’s exact test was used for categorical variables. Comparison of neurodevelopmental outcomes between two groups were further adjusted by gestational age, high-grade IVH and PVL.

## 3. Results

During the study period, there were 60 infants with NEC and 22 infants with SIP who received surgical treatment. There were no differences in gestational age (NEC vs. SIP, 26.6 vs. 26.5 weeks, *p* = 0.695) and birth weight (NEC vs. SIP, 710 vs. 745 g, *p* = 0.917) between the two groups (Table 1). Head circumferences and z-scores for gestational age and sex at birth were comparable between the groups. There were no differences in perinatal characteristics and clinical courses such as findings of SGA, RDS, moderate-to-severe BPD, sepsis, surgically treated PDA, ROP treated with laser photocoagulation, IVH, and PVL. Postnatal days (NEC vs. SIP, 15.5 vs. 6 days, *p* < 0.001) and PMA (NEC vs. SIP, 28.6 vs. 27.6 weeks, *p* = 0.046) at operation were significantly earlier in the SIP group. Although the duration of antibiotics before surgery was longer in the surgical NEC group (8 vs. 5 days, *p* = 0.042), there were no differences in use of medications during the last 7 days before surgery such as antibiotics, hydrocortisone, and inotropic agents. Mortality before discharge was slightly more prevalent in the surgical NEC group, but the difference was not statistically significant (18.3% vs. 4.6%, *p* = 0.166).

**Figure 1:**
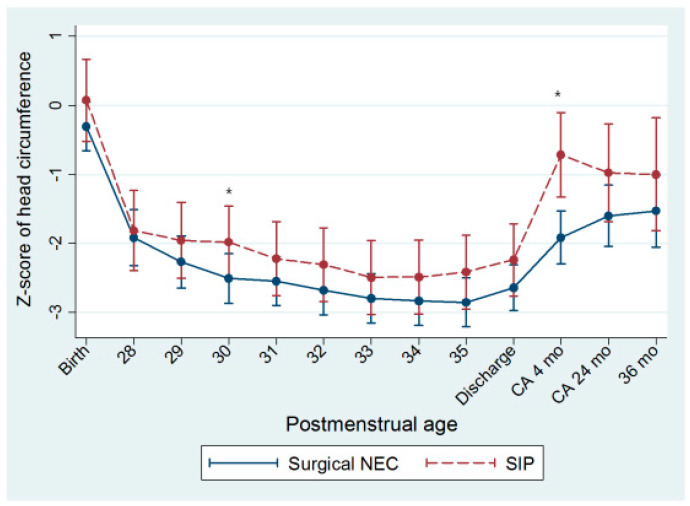
Head growth of preterm infants with surgical necrotizing enterocolitis and spontaneous intestinal perforation. Head growth at 4 months of corrected age was better in preterm infants with SIP. * Indicates *p*-value < 0.05. CA, corrected age; NEC, necrotizing enterocolitis; SIP, spontaneous intestinal perforation.

The z-score of head circumference decreased until 34 weeks PMA and was nadir at that time (Figure 1). Z-scores of weights and head circumference were comparable at PMA 34 weeks, CA 24 months, and 36 months of age (Table 2). At CA 4 months, the z-scores of head circumferences were significantly lower in the surgical NEC group (NEC vs. SIP, −2.23 vs. −0.53, *p* = 0.018). Z-score change in head circumference was higher in the SIP group than in the surgical NEC group from PMA 34 weeks to CA 4 months (NEC vs. SIP, 1.2 vs. 2.1, *p* = 0.025), while weight and growth velocities of weight were comparable at each period (Table 3).

At 24 months of age, neurodevelopmental outcomes were comparable between the surgical NEC and SIP groups (Table 4). Although combined NDI (51.2% vs. 35%, *p* = 0.284) and combined NDI or death (58.1% vs. 45%, *p* = 0.418) were slightly higher in the surgical NEC group, the difference was not statistically significant. At 36 months, abnormal findings in the relevant domains of the K-ASQ and K-DST were significantly more common in the surgical NEC group, including gross motor (48.2% vs. 0%, *p* = 0.015), fine motor (40.7% vs. 0%, *p* = 0.037), cognitive domain (55.6% vs. 12.5%, *p* = 0.047), and social domain (44.4% vs. 0%, *p* = 0.032). After adjusted for gestational age, IVH ≥ grade 3, and periventricular hemorrhage, those differences remained statistically significant.

## 4. Discussion

In the present study, there was more developmental delay at 36 months of age in preterm infants with surgical NEC than in those with SIP. Although the growth of weight and HC were comparable at CA 24 and 36 months of age between the two groups, better head growth was found at CA 4 months in the SIP group than in the surgical NEC group. In studies in the literatures, catch-up growth of head circumference in preterm infants has often been observed by CA 4 months [25,26]. We speculated that brain injury associated with surgical NEC might interfere catch-up growth of preterm infants by corrected age 4 months [15,27].

Growth of the head circumference is an important factor for neurodevelopment in preterm infants. It was also demonstrated that preterm infants with developmental delay had lower z-scores of HC at CA 4 months and 36 months in our study (Supplementary Table S1). At 5.5 years of age, preterm infants born with a birth weight < 750 g who experience a catch-up growth of the head circumference are associated with better cognitive outcomes [28]. The association between head growth and neurodevelopmental outcomes was also found in school-age children who were born as very preterm infants [29,30]. However, most preterm infants experience extrauterine growth restriction, which sometimes includes a restricted growth of head circumference [31]. Restriction and catch-up of head growth are associated with various neonatal morbidities in preterm infants. In a study that investigated the association between ROP and head growth in preterm infants who were born at less than 32 weeks of gestation, the z-score of head circumferences were lowest at around 30 weeks of PMA for infants with severe ROP, followed by catch-up growth [32]. Data from the National Institute of Child Health and Human Development (NICHD) Neonatal Research Network showed that among preterm infants with a birth weight of 1000 g or less, those with sepsis and/or NEC were more likely to have adverse neurodevelopmental outcomes, accompanied by impaired head growth [33]. The association of inflammatory conditions and growth in preterm infants was also effectively demonstrated in a prospective study of 192 very low birth weight infants, showing that infants with sustained neonatal inflammation such as sepsis, NEC, and BPD had low z-scores of head circumference, weight, and height at term, 4 months, and 12 months of CA [34].

In the present study, the lowest z-score of head circumference was observed at 34 weeks of PMA in both the surgical NEC and SIP groups, but thereafter, catch-up growth was faster in the SIP group until CA 4 months, resulting in larger head circumference in the SIP group at CA 4 months. As weight and velocity of weight gain were comparable between the two groups in this period, the impact of surgical NEC on head growth as compared with SIP, could be highlighted. This finding is intriguing because, although both SIP and surgical NEC require surgical intervention, systemic inflammation is more common in preterm infants with surgical NEC than in those with SIP, which is associated with white matter injury in preterm infants [15,35].

Subsequently, developmental delay was found more frequently in the surgical NEC group at 36 months in this study. Two previous studies from NICHD reported that the neurodevelopment of preterm infants with SIP was comparable with that of surgical NEC [11,12]; however, even though the GA and birth weights were comparable with those in the present study, higher mortality was reported in those studies. Mortality was as high as 53–59% in the surgical NEC group and 39–53% in the SIP group as compared with the 18.6% in the surgical NEC group and 4% in the SIP group in the present study. Although the higher survival rate in the current study cannot be definitively explained yet, it may be attributed to several factors, including the fact that the study period is more recent in the current study and that there are racial/ethnic differences in outcomes of NEC [36,37]. A retrospective study that reported better neurodevelopmental outcomes in the SIP also showed lower mortality of surgical NEC (28%) and SIP (22%) than the previous two studies. Moreover, this study also reported that more infants in the surgical NEC group had suboptimal head growth at 1 year of age [13].

Our study has several limitations. This was a single-center study with limited cases of surgical NEC and SIP. For the evaluation of developmental outcomes at 36 months, we summarized the results from the relevant domains in each tool arbitrarily, since either K-ASQ or K-DST was used during the study period while Bayley-III was not included in the regular checkups of preterm infants at 36 months, and only half of the survivors were tested at 36 months of age. However, demographic and growth findings were comparable between patients with and without K-ASQ/K-DST (Supplementary Tables S2 and S3). Although K-ASQ and K-DST are tools based on parental reports, they showed good agreement with other tools including BSID-II and BSID-III [38,39]. Moreover, as infants get older, the results of questionnaire-based tools by parental reporting show better agreement with the results of examiner-led tests [40,41]. Another limitation of the present study was that there were limited data associated with neurodevelopmental outcomes of preterm infants such as nutrition, dysglycemia, and growth and developmental care in NICU [42,43,44,45]. However, the aim of the present study was not to find whether SIP or surgical NEC were independent risk factors for adverse neurodevelopment, but to explore whether there were differences in neurodevelopmental outcomes among preterm infants who experienced SIP or surgical NEC.

## 5. Conclusions

In this study, catch-up growth of head circumference was faster in the SIP group until 4 months CA, followed by favorable development outcomes at 36 months of age. Inflammatory conditions in surgical NEC might have disturbed the catch-up growth of head circumference and led to delayed neurodevelopment at 36 months. More studies evaluating the neurodevelopmental outcomes of preterm infants with surgical NEC and SIP at later periods of life, such as school age and young adults, are needed.

## Figures and Tables

**Table 1:** Perinatal and neonatal characteristics of the study population.

	Surgical NEC (*n* = 60)	SIP (*n* = 22)	*p*-Value
Gestational age (weeks)	26.6 (24.3–27.5)	26.5 (25.1–27.3)	0.695
Birth weight (grams)	710 (606.0–980.0)	745 (620.0–920.0)	0.917
Birth weight z-score	−0.1 (−1.1–0.4)	−0.7 (−1.4–0.5)	0.867
Birth HC z-score	−0.2 (−1.2–0.4)	−0.1 (−1.1–0.5)	0.617
C/S	31 (51.7)	11 (50.0)	1.000
Female	23 (38.3)	9 (40.9)	1.000
Multiple birth	32 (53.3)	10 (45.5)	0.621
Outborn	22 (36.7)	8 (36.4)	1.000
Antenatal steroid use	17 (85.0)	57 (79.2)	0.534
SGA	18 (30.0)	9 (40.9)	0.429
RDS	52 (86.7)	18 (81.8)	0.725
Moderate-to-severe BPD	37 (74.0)	12 (57.1)	0.173
Sepsis	35 (59.3)	8 (36.4)	0.082
PDA operation	25 (45.5)	9 (40.9)	0.802
ROP operation	26 (53.1)	8 (38.1)	0.303
IVH ≥ grade 3	11 (18.6)	4 (18.2)	1.000
PVL	13 (23.6)	3 (13.6)	0.535
Postnatal days at surgery	15.5 (9.0–30.0)	6 (3.0–10.0)	<0.001
PMA at surgery (weeks)	28.6 (26.7–31.1)	27.6 (26.7–28.6)	0.046
PMA at discharge (weeks)	42.0 (38.5–45.29)	41.3 (39.0–43.1)	0.766
Antibiotics before surgery (days)	8 (4–17)	5 (2–7)	0.042
Last 7 days before surgery			
Hydrocortisone	6 (10)	3 (13.6)	0.696
PDA medical treatment	11 (18.3)	4 (18.2)	1.000
Inotropic agents	6 (10)	3 (13.6)	0.696
Death/s	11 (18.3)	1 (4.6)	0.166

Values are expressed as N (%) or median (interquartile range). NEC, necrotizing enterocolitis; SIP, spontaneous intestinal perforation; HC, head circumference; C/S, Cesarean section; SGA, small for gestational age; RDS, respiratory distress syndrome; BPD, bronchopulmonary dysplasia; PDA, patent ductus arteriosus; ROP, retinopathy of prematurity; IVH, intraventricular hemorrhage; PVL, periventricular leukomalacia; PMA, postmenstrual age.

**Table 2:** Growth of preterm infants with surgical necrotizing enterocolitis and spontaneous intestinal perforation.

	Surgical NEC (*n* = 60)	SIP (*n* = 22)	*p*-Value
At PMA 34 weeks			
weight z-score	−1.8 (−2.4–−1.4)	−1.9 (−2.6–−1.3)	0.662
HC z-score	−2.9 (−3.6–−1.9)	−2.6 (−3.2–−1.9)	0.262
CA 4 months			
weight z-score	−1.8 (−3.1–−0.9)	−0.9 (−2.8–−0.2)	0.089
HC z-score	−2.2 (−3.3–−0.79)	−0.5 (−1.7–0.5)	0.018
CA 24 months			
weight z-score	−1.2 (−1.9–−0.5)	−0.4 (−1.8–0.1)	0.281
HC z-score	−1.4 (−2.3–−0.4)	−0.9 (−2–0.4)	0.234
36 months			
weight z-score	−1.3 (−2.3–−0.6)	−0.9 (−2.7–−0.3)	0.856
HC z-score	−1.3 (−2.1–−0.9)	−0.9 (−2.2–−0.3)	0.421

Values are expressed as N (%) or median (interquartile range). NEC, necrotizing enterocolitis; SIP, spontaneous intestinal perforation; PMA, postmenstrual age; HC, head circumference; CA, corrected age.

**Table 3:** Growth velocity of preterm infants with surgical necrotizing enterocolitis and spontaneous intestinal perforation.

	Surgical NEC (*n* = 60)	SIP (*n* = 22)	*p*-value
Weight z-score change			
Birth to PMA 34 weeks	−1.5 (−2–−0.9)	−1.5 (−1.9–−1.0)	0.896
PMA 34 weeks to CA 4 months	0.2 (−1–1.0)	1.1 (−0.1–1.6)	0.052
CA 4 months to 24 months	0.6 (−0.3–1.9)	−0.2 (−0.8–0.8)	0.149
HC z-score change			
Birth to PMA 34 weeks	−2.3 (−3–−1.3)	−1.9 (−2.6–−1.5)	0.689
PMA 34 weeks to CA 4 months	1.2 (0.1–1.9)	2.1 (1.1–2.7)	0.025
CA 4 months to 24 months	0.3 (0.0–0.6)	−0.4 (−0.6–0.2)	0.154

Values are expressed as N (%) or median (interquartile range). NEC, necrotizing enterocolitis; SIP, spontaneous intestinal perforation; PMA, postmenstrual age; CA, corrected age; HC, head circumference.

**Table 4:** Neurodevelopmental outcomes of the study population at corrected age 24 months and 36 months.

	Surgical NEC (*n* = 49)	SIP (*n* = 21)	*p*-Value	Adjusted *p*-Value *
At CA 24 months	*n* = 42	*n* = 20		
CP	7 (16.7)	2 (10.0)	0.705	0.860
Hearing impairment	5 (11.9)	1 (5.3)	0.655	0.649
Blindness	3 (7.1)	0 (0.0)	0.156	0.110
Bayley-III (CA 18–24 months)	*n* = 31	*n* = 13		
CA at exam (months)	19.5 (18.8–22.5)	20.3 (19.2–21.7)	0.495	
Cognitive	85 (70–95)	85 (80–90)	0.979	
Language	77 (68–91)	79 (77–86)	0.486	
Motor	85 (70–91)	88 (79–100)	0.366	
Cognitive & language < 85	12 (38.7)	5 (38.5)	1.000	0.654
Motor < 85	15 (48.4)	5 (38.5)	0.742	0.889
Development delay in Bayley-III	16 (51.6)	6 (46.2)	1.000	0.915
NDI	22 (51.2)	7 (35.0)	0.284	0.347
Death or NDI	25 (58.1)	9 (45.0)	0.418	0.189
At 36 months				
K-ASQ or K-DST	*n* = 27	*n* = 8		
Language < cut-off	14 (51.9)	1 (12.5)	0.101	0.083
Gross motor < cut-off	13 (48.2)	0 (0.0)	0.015	0.012
Fine motor < cut-off	11 (40.7)	0 (0.0)	0.037	0.043
Cognitive < cut-off	15 (55.6)	1 (12.5)	0.047	0.026
Social < cut-off	12 (44.4)	0 (0.0)	0.032	0.016
Any < cut-off	16 (59.3)	2 (25)	0.121	0.077

Values are expressed as N (%) or median (interquartile range). NEC, necrotizing enterocolitis; SIP, spontaneous intestinal perforation; CA, corrected age; CP, cerebral palsy; Bayley-III, Bayley Scales of Infant and Toddler Development 3rd Edition; NDI, neurodevelopmental impairment; K-ASQ, Korean Ages and Stages Questionnaire; K-DST, Korean Developmental Screening Test. * Adjusted for gestational age, intraventricular hemorrhage ≥ grade 3, and periventricular hemorrhage.

## Data Availability

The datasets generated and analyzed are not publicly available but are available from the corresponding author on reasonable request.

## Supplementary Materials

The following are available online at https://www.mdpi.com/article/10.3390/children8100833/s1, Table S1: Characteristic of study population according to the developmental delay in K-ASQ/K-DST, Table S2: Perinatal and neonatal characteristics of preterm infants with or without K-ASQ/K-DST, Table S3: Growth of preterm infants with or without K-ASQ/K-DST.
