# Supplementary material for: Head Growth and Neurodevelopment of Preterm Infants with Surgical Necrotizing Enterocolitis and Spontaneous Intestinal Perforation
Source: Children (Basel). 2021 Sep 23;8(10):833. doi: 10.3390/children8100833 (PMC8534747; doi:10.3390/children8100833)
Supplement: Supplementary file 1 [file children-08-00833-s001.zip › children-1357293-supplementary.pdf]

Table S1. Characteristic of study population according to the developmental delay in K-ASQ/K-DST

	Normal K-ASQ/K-DST (n=17)	Any abnormal findings in K-ASQ/K-DST (n=18)	p value
GA (week)	27.3 (26.9-27.9)	24.4 (23.9-27)	0.002
Birth weight (gram)	920 (670-1020)	682 (540-790)	0.033
Birth weight z-score	0 (-1-0.2)	0 (-1-0.5)	0.692
Female	9 (52.9)	6 (33.3)	0.315
HC z-score at birth	-0.1 (-1.1-0.5)	-0.2 (-1.6-0.5)	0.695
HC z-score at CA 4 months	0.1 (-1-1)	-2.9 (-3.8--2.1)	0.000
HC z-score at 36 months	-0.8 (-1.3-0)	-2 (-2.6--1.2)	0.028
Moderate to severe BPD	10 (58.8)	15 (83.3)	0.146
IVH ≥ grade 3	1 (5.9)	4 (22.2)	0.338
PVL	1 (5.9)	6 (33.3)	0.088

Values are expressed as N (%) or Median (interquartile range). K-ASQ/K-DST, Korean Ages and Stages Questionnaire or Korean Developmental Screening Test; GA, gestational age; HC, head circumference; C/S, BPD, bronchopulmonary dysplasia; IVH, intraventricular hemorrhage; PVL, periventricular leukomalacia

Table S2. Perinatal and neonatal characteristics of preterm infants with or without K-ASQ/K-DST

	K-ASQ/K-DST (-) (n=47)	K-ASQ/K-DST (+) (n=35)	p value
GA (week)	26.4 (24.4–27.3)	26.9 (24.3–27.6)	0.500
Birth weight (gram)	700 (570–970)	760 (630–1000)	0.518
Birth weight z-score	-0.5 (-1.4–0.5)	0 (-1–0.3)	0.711
Birth HC z-score	-0.3 (-1.2–0.4)	-0.2 (-1.1–0.5)	0.802
C/S	26 (55.3)	16 (45.7)	0.503
Multiple birth	23 (48.9)	19 (54.3)	0.661
Outborn	20 (42.6)	10 (28.6)	0.249
Antenatal steroid	32 (80)	25 (78.1)	1.000
SGA	16 (34)	11 (31.4)	1.000
RDS	42 (89.4)	28 (80)	0.344
Moderate to severe BPD	24 (66.7)	25 (71.4)	0.798
Sepsis	26 (56.5)	17 (48.6)	0.508
PDA operation	18 (42.9)	16 (45.7)	0.802
ROP operation	17 (48.6)	17 (48.6)	1.000
IVH ≥ grade 3	10 (21.7)	5 (14.3)	0.565
PVL	9 (21.4)	7 (20)	0.535

Values are expressed as N (%) or Median (interquartile range). K-ASQ/K-DST, Korean Ages and Stages Questionnaire or Korean Developmental Screening Test; GA, gestational age; HC, head circumference; C/S, Cesarean section; SGA, small for gestational age; RDS, respiratory distress syndrome; BPD, bronchopulmonary dysplasia; PDA, patent ductus arteriosus; ROP, retinopathy of prematurity; IVH, intraventricular hemorrhage; PVL, periventricular leukomalacia

Table S3. Growth of preterm infants with or without K-ASQ/K-DST

	K-ASQ/K-DST (-) (n=47)	K-ASQ/K-DST (+) (n=35)	p value
At PMA 34 weeks			
weight z-score	-1.9 (-2.4 – -1.2)	-1.8 (-2.2 – -1.4)	0.886
height z-score	-2.6 (-3.3 – -1.6)	-2.3 (-3.3 – -1.6)	0.714
HC z-score	-2.9 (-3.5 – -1.9)	-2.6 (-3.5 – -1.9)	0.962
CA 4 months	n=31	n=32	
weight z-score	-1.5 (-3.1 – -0.8)	-2.0 (-2.8 – -0.3)	0.984
height z-score	-2.1 (-3.0 – -0.5)	-1.8 (-3.5 – -0.8)	0.789
HC z-score	-2.0 (-2.5 – -0.1)	-1.4 (-3.1– 0.1)	0.924
CA 24 months	n=29	n=34	
weight z-score	-1.3 (-1.8 – -0.4)	-1.1 (-1.7 – -0.3)	0.772
height z-score	-1.0 (-1.7 – -0.4)	-0.9 (-1.9 – -0.1)	0.567
HC z-score	-1.2 (-2.1 – -0.3)	-1 (-2.2 – -0.4)	0.923
36 months	n=23	n=29	
weight z-score	-1.9 (-2.4 – -0.4)	-1.2 (-2.3 – -0.3)	0.333
height z-score	-1.7 (-2.4 – -1.3)	-1.2 (-2.2 – -0.4)	0.148
HC z-score	-1.2 (-2.2 – -0.7)	-1.2 (-2.2 – -0.4)	0.918

Values are expressed as N (%) or Median (interquartile range). K-ASQ/K-DST, Korean Ages and Stages Questionnaire or Korean Developmental Screening Test; PMA, postmenstrual age; HC, head circumference; CA, corrected age
